# A systematic review and meta-analysis of the impact of environmental disadvantage on youth delayed reward discounting

**DOI:** 10.1017/S0954579425100886

**Published:** 2025-11-12

**Authors:** Julia W. Felton, Geoffrey Kahn, Jaclyn Johnson, Hira Ali, Souad Saleh, Nadya Habib, Brion Maher, Justin C. Strickland, JeeWon Cheong, Richard Yi, Jill A. Rabinowitz

**Affiliations:** 1 Center for Health Policy & Health Services Research, Henry Ford Health, Detroit, MI, USA; 2 College of Human Medicine, Michigan State University, East Lansing, MI, USA; 3 Department of Mental Health, Bloomberg School of Public Health, Johns Hopkins University, Baltimore, MD, USA; 4 Department of Psychiatry and Behavioral Sciences, Johns Hopkins University School of Medicine, Baltimore, MD, USA; 5 Center for Behavioral Economic Health Research and Department of Health Education and Behavior, College of Health & Human Performance, University of Florida, Gainesville, FL, USA; 6 Cofrin Logan Center for Addiction Research and Treatment and Department of Psychology, University of Kansas, Lawrence, KS, USA; 7 Department of Psychiatry, Robert Wood Johnson Medical School, Rutgers University, Piscataway, NJ, USA

**Keywords:** Adolescents, adversity, children, delay discounting, environment, stress

## Abstract

Delayed reward discounting (DRD), the tendency to prefer smaller rewards available immediately relative to larger rewards available after a delay, is associated with numerous health outcomes across the lifespan. Emerging literature points to the central role of early environments, specifically factors reflecting harshness (including lack of resources) and unpredictability (exposure to instability and stressful events) in the development of DRD. Yet, existing research uses disparate indicators of environmental risk and often draws on small samples resulting in conflicting findings, making comparisons across studies challenging. The current systematic review examined environmental factors that may place youth at greatest risk for heightened DRD and subsequent negative health outcomes. Search results identified 28 articles reflecting 20 unique samples. Additionally, meta-analyses were conducted to examine overall effects for the two most commonly examined environmental predictors (family income and family history of substance use disorder). Results suggest small-to-medium associations of environmental risk with DRD, with smaller associations observed for more distal predictors of harshness (e.g., family income) and larger associations among more proximal indicators of environmental instability (e.g., harsh parenting and parental pathology). Findings highlight the role of environmental factors on DRD development and may inform future interventions.

## Introduction

Decision-making tendencies that favor smaller rewards available immediately relative to larger rewards after a delay (known as *delayed reward discounting,* DRD) have been found to be associated with a number of maladaptive health outcomes across the lifespan. Among adults, steeper rates of delay discounting, reflecting a stronger preference for smaller but immediately available rewards, have been linked to greater rates of obesity (Bickel et al., 2021), substance use disorder (MacKillop et al., 2011), and gambling (Weinsztok et al., 2021); although some have described these associations as small and/or non-disorder-specific effects (see review in Bailey et al., 2021; see also Stein et al., 2023 for opposing view point). Emerging evidence suggests that the negative impacts of DRD may be apparent even earlier in development. For example, steeper discounting predicts more rapid escalation (Felton et al., 2020) and more problematic patterns of substance use (Cassidy et al., 2020) among children and adolescents. Additionally, steeper DRD has been found to be associated with greater risk for childhood obesity (Tang et al., 2019), higher rates of problematic gambling (Secades-Villa et al., 2016), and greater severity of internet addiction (Qi et al., 2024), as well as worse outcomes in treatment for behavioral addictions (Stanger et al., 2012) during this developmental period.

Decision-making tendencies that favor smaller rewards available immediately relative to larger rewards after a delay (known as *delayed reward discounting,* DRD) have been found to be associated with a number of maladaptive health outcomes across the lifespan. Among adults, steeper rates of delay discounting, reflecting a stronger preference for smaller but immediately available rewards, have been linked to greater rates of obesity (Bickel et al., 2021), substance use disorder (MacKillop et al., 2011), and gambling (Weinsztok et al., 2021); although some have described these associations as small and/or non-disorder-specific effects (see review in Bailey et al., 2021; see also Stein et al., 2022 for opposing view point). Emerging evidence suggests that the negative impacts of DRD may be apparent even earlier in development. For example, steeper discounting predicts more rapid escalation (Felton et al., 2020) and more problematic patterns of substance use (Cassidy et al., 2020) among children and adolescents. Additionally, steeper DRD has been found to be associated with greater risk for childhood obesity (Tang et al., 2019), higher rates of problematic gambling (Secades-Villa et al., 2016), and greater severity of internet addiction (Qi et al., 2024), as well as worse outcomes in treatment for behavioral addictions (Stanger et al., 2012) during this developmental period.

Given the negative public health implications of DRD, identification of early and potentially malleable indicators of risk for steeper discounting provides a target for novel prevention and intervention efforts. Although numerous studies have examined the role of a variety of early environmental exposures in predicting youth discounting, there has not been, to our knowledge, a comprehensive review of these relations. To address this gap, the current study conducted a systematic review and a meta-analysis to evaluate associations between environmental factors and youth rates of DRD.

Life history theory suggest that exposure to harshness (including lack of socioeconomic resources) and unpredictability (including traumatic and other stressful events) in the environment shapes decision-making and, subsequently, health outcomes (Roff, 1993; 2002). Decision-making focused on obtaining immediate rewards would, according to this framework, be more adaptive in environments characterized by instability and limited access to resources. In other words, harsh and unpredictable environments would favor steeper rates of delay discounting.

In support of this theory, research has found that individuals raised in low-resource environments demonstrate a preference for “smaller sooner” rewards into adulthood with subsequent impacts on health outcomes (Griskevicius et al., 2011). For instance, one study found that adults who reported greater adverse experiences in childhood evidenced greater delay discounting and a higher body mass index (Lovallo et al., 2013). Another study found that adults who perceived their childhood as unpredictable had both steeper rates of delay discounting and engaged in more externalizing behaviors (Martinez et al., 2022). Thus, studying environmental exposures during childhood, which may be a critical developmental period during which decision-making becomes stable and likely to be sustained into adulthood (Simpson et al., 2012), is central to mapping trajectories of decision-making across the lifespan.

While the extant literature points to the effects of early environmental disadvantage on development of DRD, the extent to which these constructs are associated with youth DRD across the literature has not been reviewed. Understanding the associations of harsh and unpredictable environments on decision-making is critical to guiding policy and supporting targeted early life interventions. The goals of this systematic review and meta-analysis are to summarize the current state of the literature and quantify the strengths of associations between environmental disadvantage, operationalized utilizing life history frameworks including harshness (lack of access to resources) and unpredictability (exposure to traumatic and stressful events), and DRD among youth. Given that the preponderance of literature has operationalized environmental harshness as family-level indicators of income, we also conducted a meta-analysis to examine the specific association of income variables with delay discounting.

## Methods

This systematic review follows the Preferred Reporting Items for Systematic Reviews and Meta-Analyses (PRISMA; Moher, 2009) and the protocol was preregistered at PROSPERO (Schiavo, 2019): https://www.crd.york.ac.uk/prospero/display_record.php?RecordID=467088. An evaluation of study quality was completed by three authors (JJ, HA, SS) using the NHLBI’s Quality Assessment Tool for Observational Cohort and Cross-Sectional Studies. Results were then discussed with a fourth author (JF) to reach consensus in the case of any disagreements.

### Eligibility criteria

All studies were screened to ensure they met the following inclusion criteria, including that each study (1) utilized human subjects; (2) included a quantitative measurement of at least one specific environmental indicator of disadvantage or adversity; (3) included a quantitative measure of monetary delay discounting or intertemporal choice; (4) examined the statistical relation (cross-sectional or longitudinal) between the environmental indicator and delay discounting; and (5) was written in English and published in peer-reviewed journals through 2023. Results were then screened to identify studies where relations could be specifically examined for youth under 18 years old. Additionally, only quantitative studies (rather than qualitative research) were included. In the case of studies that conducted interventions or subjected participants to experimental paradigms, only baseline data (prior to intervention) on the environment and DRD were examined. No restrictions were made based on clinical conditions of participants.

### Information sources and search strategy

The development of the search strategy and initial literature search were supported by health sciences librarians at Henry Ford Health. Searches were conducted using the following databases: PubMed, PsycINFO (EBSCO), and Google Scholar. The searches were based on a combination of keyword terms and controlled vocabulary related to “early environmental exposure” and “delay discounting.” Exact search terms are included in the Supplementary Materials. The search was conducted originally in October 2023 and updated in May 2025 and included papers published between 01/01/1990 and 12/31/2024. A second health sciences librarian peer-reviewed the searches. Results were then imported into the Covidence systematic review management software (https://www.covidence/org/) to support removal of duplicate sources and review of study data. Additionally, reference sections of relevant articles were searched to identify additional publications that may have been missed in the original search.

**Figure 1.**
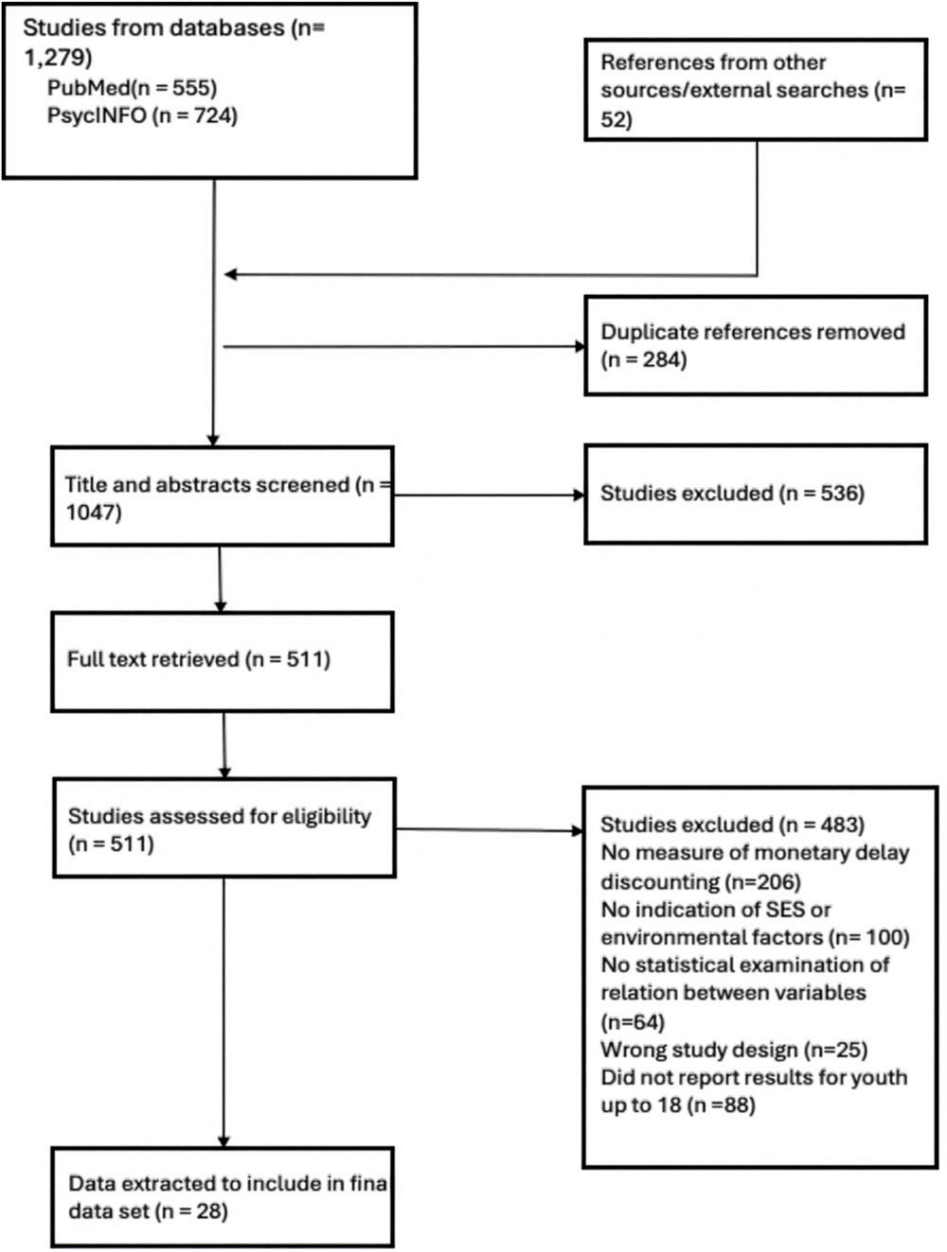
PRISMA flow diagram.

Data screening was done by three of the authors (JJ, HA, SS) in two stages within Covidence. First, titles and abstracts were reviewed to determine whether they met the inclusion criteria (see Figure 1), and ineligible studies were removed from the search. Second, a full-text review of the identified articles was conducted to confirm inclusion and identify any additional papers through a search of relevant bibliographies. A fourth author (NH) independently reviewed all full texts to ensure agreement. Discrepancies were resolved through discussion with a PhD-level clinical psychologist (JF).

### Data extraction

Data from the final set of included articles was extracted in Covidence by two independent authors (JF and GK). Extracted information included sample size, sample demographic data (age, sex, race/ethnicity), measurement descriptions, and reported associations between measures of environmental disadvantage and delay discounting. Extracted data was compared, and any discrepancies were discussed until consensus was reached.

### Data synthesis and meta analysis

Characteristics of the final set of included studies are shown in Table 1. A meta-analysis was conducted to examine the overall pooled effect of family-level indicators of income (the most widely utilized index of environmental disadvantage) and DRD using MetaXL (Barendregt & Doi, 2016), an extension for Microsoft Excel. Standardized relations between family income indicators were computed for each study. For a study where a bivariate association between these constructs was not available (Crandall et al., 2021), we contacted the authors and obtained the correlation. Correlations were z-transformed prior to modeling, though the results presented are back-transformed. Funnel plots were also examined to detect potential publication or other systematic biases. Heterogeneity among studies was also examined using both the *Q* statistic (which tests the statistical significance of heterogeneity between studies; significance threshold set at *p* < 0.05) and the *I* ^2^ statistic (which indicates the percentage of variation between studies due to heterogeneity rather than chance). If heterogeneity was found, *post hoc* sensitivity analyses would be conducted to identify individual studies that may contribute to heterogeneity.

**Table 1.** Summary of findings

Authors, Year	Sample Size	Sex (% Males)	Race/Ethnicity	Age (Years)	Measure and Index of DRD	Adjusting?	Measure of Environment	Study Design	Sample	Summary of Results
Anil et al., 2011	866	48.7%	46.6% White, 47.5% Black,	Median: 13.8	Study developed measure	No	Having both parents in household, parent/guardian educational attainment, parent/guardian employment status, if family receives child support, if family receives government assistance, if family uses short-term financial services, if family received government housing assistance, if family had ever been evicted	Observational; cross-sectional	American community	Being evicted and not having both parents in the house were associated with steeper DRD
Borissova et al., 2022	76	50%	68% White, 20% Mixed, 2.7% Asian, 5.3% Black, 4% Other,	Mean: 17.10	MCQ (*k*) (Kirby et. al, 1999)	No	Maternal education	Observational; longitudinal but data taken from Time 1 youth who use cannabis	English adolescents who used cannabis	No significant relation between maternal education and child DRD
Bridge et al., 2015	80	25%	80% White	Mean: 15.6	Delay Discounting Task (AUC) (Richards et al., 1999)	Yes	Family history of suicide	Observational; cross-sectional	American children who had attempted suicide and children who had psychiatric disorders	Among youth in with a family history of suicidal behavior there was no relation between family history of suicide behavior and DRD; among youth with other psychiatric disorders, greater family history of suicidal behaviors were associated with steeper rates of DRD
Button et al., 2023	411	47.9%	67.9% White	Mean: 9.4	Adjusting Delay Discounting Task (AUC) (Du, Green & Myerson, 2002).	Yes	Parent educational attainment, combined family income and Medicaid status	Clinical Trial; data taken from baseline	American children of parents with obesity	Non-significant relations between parent educational attainment, Medicaid status and family income with child DRD
Crandall et al., 2021	243	49.4%	African American 11.9%, White 76.5%, Other/ more than one race 11.1%, Hispanic or Latinx 7%	Mean: 13	Adjusting Amount Delay Discounting Task (k) (Johnson & Bickel, 2002).	Yes	Parent/guardian marital status, employment status, education, total household income, employment income, government assistance, child support/alimony, disability and occupation. Parents/guardians answered questions about their own feelings of food insecurity as well as the overall household food insecurity levels using a 17-item subset of the USDA Household Food Security scale.	Observational; longitudinal	American community	Lower rates of parent/guardian education, occupational prestige and higher level of household food insecurity were associated with steeper DRD
DeRosa et al., 2024	1843	66.1%	African American 11.44%, White 48.49%, Hispanic or Latinx 24.14%, Asian/Pacific Islander 2.96%, Other/more than one race 12.97%	Mean: 10.6	Adjusting Delay Task-5 items (*k*) (Johnson & Bickel, 2002).	Yes	Total household income	Observational; cross-sectional	American community	Lower household income associated with steeper DRD
Dougherty et al., 2014 ^a^	386	48.4%	10.9 % African-American, 86.8 % Caucasian, 2.3% Other Ethnicity: 78.4% Hispanic/Latino, 21.5% Non-Hispanic/Latino	Median: 11.49	MCQ (*k*) (Kirby et. al, 1999)	No	Four Factor Index of Socioeconomic Status; Family History of SUD	Observational; longitudinal, data from first wave with available measures	American family history of SUD	Family history of SUD associated with steeper DRD
Dougherty et al., 2015 ^a^	386	48.4%	10.9 % African-American, 86.8 % Caucasian, 2.3% Other Ethnicity: 78.4% Hispanic/Latino, 21.5% Non-Hispanic/Latino	Median: 11.49	MCQ (*k*) (Kirby et. al, 1999)	No	Four Factor Index of Socioeconomic Status; Family History of SUD	Observational; longitudinal	Family history of SUD	No significant differences in trajectories of DRD over time based on family history of DRD
Farley & Kim-Spoon, 2017 ^c^	220	55%	89% White, 11% African American, Hispanic, or other races.	Mean: 13.03	MCQ (*k*) (Kirby et al., 1999)	No	Parent marital status, household income, financial satisfaction, financial worry, and how well off they perceive themselves to be	Observational; longitudinal	American community	Steeper rates of DRD related to: income, income satisfaction, perceived financial status, financial worry. Latent SES factor was not significantly related to DRD
Felton et al., 2020 ^d^	247	56%	52.5% White, 37.7% Black, 1.6% Asian, 8.2% other race	Mean: 13	MCQ (*k*) (Kirby et al., 1999).	No	Household income	Observational; longitudinal	American community	Lower family income associated with steeper rates of DRD
Felton et al., 2021 ^d^	277	56.4%	52.7% White, 37.9% Black, 9.4% Other Race/Ethnicity	Mean: 13.06	MCQ (*k*) (Kirby et al., 1999)	No	Maternal depressive symptoms; maternal marijuana use	Observational; longitudinal	American community	Baseline maternal depressive symptoms not significantly related to baseline youth DRD, but significantly related to youth DRD at follow-up; baseline maternal marijuana use not related to youth DRD
Felton et al., 2022 ^d^	213	55%	53% White, 38% African American, 2% Hispanic, 2% Asian, 8% Other Race/Ethnicity	Mean: 15.02	MCQ (*k*) (Kirby et al., 1999)	No	Coddington’s Life Events Questionnaire ; Family socioeconomic status	Observational; longitudinal	American community	Lower socioeconomic status was associated with steeper DRD
Felton et al., 2024 ^d^	246	53.3%	52.5% White, 35.7% Black/African American, 11.8% Other Race/Ethnicity	Mean: 11.96	MCQ (*k*) (Kirby et al., 1999)	No	Socioeconomic distress index, including census-derived indicators: low educational attainment, lone parenthood, low income and unemployment status; perceived environmental supports	Observational; longitudinal	American community	Socioeconomic distress was associated with DRD cross-sectionally and longitudinally; perceived environmental support was interacted with socioeconomic distress to predict changes in DRD over time
Henderson et al., 2018	188	50.1%	Not reported	Mean: 15.5	Delay Discounting Task (*k*) (Mitchell, 1999)	No	Family history of SUD	Observational; cross-sectional	American family history of SUD	Participants with a family history of SUD demonstrated steeper rates of DRD
Herting et al., 2010	33	51.5%	90.9% White	Mean: 13.8	Delay Discounting Task (*k*) (Mitchell, 1999)	No	Family history of SUD	Observational; cross-sectional	American family history of SUD	Participants with a family history of SUD demonstrated a non-significant trend towards steeper rates of DRD and had slower response times on choice trials; no differences on DRD between groups after controlling for response times
Kim-Spoon et al., 2015 ^c^	106	48%	85% White, 11% African American, 2% Hispanic, 2% Other ethnic groups	Mean: 11.59	MCQ (*k*) (Kirby et al., 1999)	No	Annual household income	Observational; longitudinal	American community	No significant relation between family income and DRD
Kim-Spoon et al., 2019 ^b^	157	52%	82% White, 12% African American, and 6% Other ethnic groups	Mean: 14.13	Computerized Delay Discounting Task (*k*) (Johnson & Bickel, 2002)	Yes	CHAOS, family “Multi-risk index” including: parents’ and their spouses education, family income, public income assistance	Observational; longitudinal	American community	Family multirisk index was associated with steeper DRD, but DRD was unrelated to CHAOS and parent education
Liu et al.,2020	101	52%	75.2% Black, 10.9% White, 8.9% Latino(a), 1% Native American, 4% Other	Range: 9-12	MCQ (*k*) (Kirby et al., 1999).	No	Harsh parenting; household income 200% below federal poverty line	Observational; longitudinal	American low-income families	Harsher parenting was associated with higher levels of DRD
Owens et al., 2022	4042	52%	72% White, 11% Mixed, 9% Black, 3% Other, Ethnicity: 78.4% Hispanic/Latino, 21.5% Non-Hispanic/Latino, 2% Asian, 1% American Indian/ Pacific Islander	Mean: 10	42-item adjusting amount (AUC) (Koffarnus & Bickel, 2014)	Yes	Household Income and Parental Marital status	Observational; longitudinal, data from first wave with available measures	American community	Parent marital status associated with steeper DRD
Paraskevopoulou et al., 2020	270	56%	Not reported	Mean: 16.39	40-trial of money available immediately or after a brief delay (5, 10, 20, 30 or 60s) (AUC) (Scheres et al., 2014)	No	Family history of SUD	Observational; cross-sectional	Dutch NeuroIMAGE cohort of youth with and without ADHD	In two-way ANCOVA, no impact of family history of SUD on rates oF DRD, controlling
Peviani et al., 2019 ^b^	167	53%	79% White, 13.8% African American, 2% Hispanic, 1.8% Asian, 0.6% American Indian/Alaska Native, 4.8% More than one Race	Mean: 14.07	Computerized Delay Discounting Task (*k*) (Johnson & Bickel, 2002)	Yes	Conflict subscale of the Parent–Child Relationship Inventory; Confusion, Hubbub and Order Scale (CHAOS); annual household income	Observational; longitudinal	American community	Harsher parenting and more household chaos associated with steeper DRD
Peviani et al., 2024a^b^	167	53%	79% White, 13.8% African American, 2% Hispanic, 1.8% Asian, 0.6% American Indian/Alaska Native, 4.8% More than one Race	Mean: 14.07	Computerized Delay Discounting Task (*k*) (Johnson & Bickel, 2002)	Yes	Socioeconomic status index, including: household income, parents years of education	Observational; longitudinal	American community	Higher socioeconomic status at Time 1 was associated with steeper rates of DRD and Time 2 and Time 4
Peviani et al., 2024b^b^	167	53%	78% White, 14% African American, 2% Hispanic, 1% Asian, 1% American Indian/Alaska Native, 6% More than one Race	Mean: 14.07	Computerized Delay Discounting Task (*k*) (Johnson & Bickel, 2002)	Yes	Maltreatment and Abuse Chronology of Exposure Scale	Observational; longitudinal	American community	No significant relations between abuse and DRD at any timepoint; significant associations between higher rates of neglect and steeper rates of DRD at Time 2-5
Reynolds et al., 2009	30	50%	Not reported	Mean: 12.5	MCQ (*k*) (Kirby et al., 1999)	No	Maternal cigarette use (15 cigarettes/day or more)	Observational; cross-sectional	American maternal cigarette use	Participants with a mother who smoked demonstrated steeper rates of DRD at small, medium and large reward magnitudes
Rodriguez-Moreno et al., 2021	125	52%	26% Black, 60% Hispanic, 14% Other Race/Ethnicity	Mean: 15.11	Binary choice task with two trial types (now/not now where the smaller reward is available immediately or after a two week delay) and framing conditions (delay/accelerated where smaller and larger rewards are alternatively presented as the default choice) (analyzed using a Bayesian model including multiple response variables) (Decker et al., 2015)	No	Family history of SUD	Observational; cross-sectional	American family history of SUD recruited from “inner-city”	Participants with a family history of SUD were less likely to choose larger delayed rewards in the “now” condition, but more likely choose larger delayed rewards in the “not now” trials, and less likely to show increases in their preferences for larger later rewards as the reward amount increased
Sheehan et al., 2024	212	41.5%	65.4% White, 31.8% Black, 8.7% Hispanic, 2.8% Asian, 1.9% American Indian or Alaska Native, .9% Native Hawaiian or Other Pacific Islander	Mean: 15.02	Computerized Delay Discounting Task (*k*) (Koffarnus & Bickel, 2014)	Yes	Parents self-injurious behavior	Observational; cross-sectional	American community	Greater parent history of self injurious behavior associated with steeper rates of DRD among youth
Stanger et al., 2012	165	87.9%	38% Caucasian, 59% African American, 2% Multiracial, 1% Native American	Mean: 15.77	4 trials with smaller and larger dollar and marijuana equivalent amounts (*k*) (Baker, Johnson, & Bickel 2003; Johnson& Bickel 2002).	Yes	Nine-step Hollingshead Index	Clinical Trial, data taken from baseline	American clinical trial for adolescent SUD	Lower SES indicators was associated with steeper DRD
Zhao et al., 2022	207	50%	Not reported (mostly Han population)	Mean: 12.7	Chinese-adapted MCQ (proportion of trials in which participant chose larger later reward) (Kirby et al., 1999)	No	Socioeconomic status index, including: material wealth, parents’ education and occupational levels, and monthly income	Observational; cross-sectional	Chinese community	Lower SES was associated with steeper rates of DRD among youth

*Note.* MCQ = Monetary Choice Questionnaire; DRD = Delayed Reward Discounting; SUD = Substance Use Disorder; AUC = Area Under the Curve. Superscripts indicate data drawn from the samples.

## Results

### Search results

The initial electronic database search identified 1,279 studies (see Figure 1). An additional 52 papers were identified during a search of bibliographies of relevant papers. Following the removal of duplicates and an initial title and abstract screen, 511 studies were identified for full-text review. Twenty-eight publications met inclusion criteria and were included in the final sample (see Table 1 for detailed characteristics of each study). Of these, eleven articles reported on results using overlapping samples, including two articles reporting on one sample (Farley & Kim-Spoon, 2017; Kim-Spoon et al., 2015), four articles reporting on a second sample (Kim-Spoon et al., 2019; Peviani et al., 2019, 2024a, 2024b), two articles reporting on a third sample (Dougherty et al., 2014, 2015), and four articles reporting on a fourth sample (Felton et al., 2020, 2021, 2022, 2024), suggesting the articles included in this review represent 20 unique samples. Because all articles using the same sample also reported on either different indicators of socioeconomic disadvantage or different outcomes, each was included in the review individually, but only one article representing each unique samples was used in meta-analyses of the effects of either family-level indicators of income, parental education, or familial substance use disorder on youth discounting. Which publication (of those using the same sample) was included in the meta-analysis was determined by selecting for the manuscript that used either the environmental characteristic most similar to other studies in the analysis or which was collected earlier (e.g., the publication reporting on baseline associations rather than those at follow-up).

### Study design and quality

Of the 20 unique samples included in the review, 18 were observational studies (including ten cross-sectional designs and eight longitudinal designs) while two reported on baseline results from clinical trials. Longitudinal designs ranged from one- to three-year follow-ups, and one included data from the Adolescent Brain Cognitive Development (ABCD) study, which is currently collecting data.

Twenty-three articles received the highest quality rating. The remaining articles received quality rating of fair. Reasons for lower quality ratings included a lack of clearly defined outcome measures, lack of effect size reporting, and attrition rates over 20%. No articles received a quality rating of poor.

### Sample characteristics

Three of the studies were from non-US samples, while the rest included only American participants. Participants for ten unique samples (represented across 17 articles) were recruited from the community (with one drawn from a low-resource community). Additionally, 11 articles reported on ten distinct clinical samples (ranging from youth with family histories of substance use disorder to youth entering treatment for SUD, youth with parents with obesity, youth with ADHD, youth who use cannabis, and youth who had previously attempted suicide and a matched comparison group with psychiatric disorders). Sample sizes ranged from 30 to 4,042 and were fairly diverse with respect to demographic factors. Percentages of participants who identified as White/Caucasian ranged from 0% to 90.9%, while percentages of participants who identified as male ranged from 25 to 87.9% of each sample. Mean ages of each sample ranged from 9.4 to 17.1 years old.

### Indicators of delayed reward discounting

As part of the inclusion criteria, all studies were required to include a monetary measure of delayed reward discounting. Of the 20 unique samples, seven utilized the Monetary Choice Questionnaire (MCQ; Kirby et al., 1999) or a version adapted for Chinese samples (Zhao et al., 2022). The MCQ is a widely used, paper-and-pencil measure originally developed for adults who misuse illicit substances. The measure asks participants to choose between a smaller monetary value available immediately or a larger value available after a delay (e.g., $15 today or $35 in 13 days). The values of the small and large rewards, as well as the length of the delay, are varied across the studies and can be divided into smaller, medium-sized, and larger amounts of the delayed reward. Two studies used the titration delay discounting task adapted by Mitchell and colleagues (Mitchell, 1999). One study (Anil et al., 2011) used a similar paper-and-pencil measure that also asked about preferences for smaller or larger amounts and was adapted by the author team. Another used a similar approach but with considerably smaller reward amounts (between 1 and 5 cents) and shorter delays (5, 10, 20, 30, or 60 s) (Paraskevopoulou et al., 2020). The final study (Rodriguez-Moreno et al., 2021) used a similar binary choice task adapted to include trial types that varied when the “smaller sooner” reward amount was available (now vs. two weeks later with further delay for larger later reward) and trial framing where the default option (denoted as the first response using a visual indicator) was either a “smaller sooner” or “larger later” reward.

All of the remaining studies (*n* = 8) administered computerized, adaptive measures that also asked participants to select between smaller and larger dollar amounts available after a delay (similar to the tasks described above). In these tasks, however, either the amount of reward or the length of delay are systematically varied based on a participant’s response to previous items.

All studies indexed rates of discounting calculated as either a *k* value or non-parametric area under the curve (AUC), with the exception of two (Rodriguez-Moreno et al., 2021; Zhao et al., 2022) that examined participant responses (e.g., proportion of larger later choices) across varying conditions of the binary choice task (choice behaviors in the context of specific manipulations of length of delay or default choice). Because indices of discounting are often skewed, computed values were typically log-transformed to create normal distributions.

### Indicators of environmental disadvantage

Indicators of environmental disadvantage varied across studies. Many studies (*n* = 8) included a specific measure of household income or a closely related concept (*n* = 4), including Hollingshead indices reflecting parents’ employment prestige/earnings and indices of socioeconomic status (including composites of wealth, parental education and other indicators). A number of studies also included factors more broadly reflective of household SES, including receipt of public assistance (*n* = 3), Medicaid enrollment (*n* = 1), parents’ perceptions of their financial status (*n* = 1), and parents’ employment (*n* = 2). Only one study in the current review included objective indicators of community-level disadvantage, including census-derived levels of low educational attainment, lone parenthood, low income, and unemployment status (Felton et al., 2024). Additionally, this was the only study to evaluate the impact of a positively-valanced indicator (perceived environmental support) on youth rates of DRD.

Other studies included parent- and parenting-related factors. These included parents’ education level (*n* = 5) and parents use of harsh or abusive parenting practices (*n* = 3). Broader household measures included indicators of household chaos (reported on in two articles using the same sample of participants), household food insecurity (*n* = 1), and housing instability (*n* = 1).

Finally, other studies included measures of established adverse childhood experiences (ACEs; Felitti, 2009), including parental divorce/marital status (*n* = 3), parents or family members using substances (*n* = 7), family history of suicide (*n* = 1) or self-injurious behavior (*n* = 1), family antisocial behaviors (*n* = 1), and parental depression (*n* = 1). Additionally, one study examined dependent and independent stressful life events (*n* = 1).

Each of these relations is reviewed below in detail and bivariate correlation coefficients are reported between indicators of environmental disadvantage and youth discounting when available.

### Associations between indicators of family income and youth discounting

**Figure 2.**
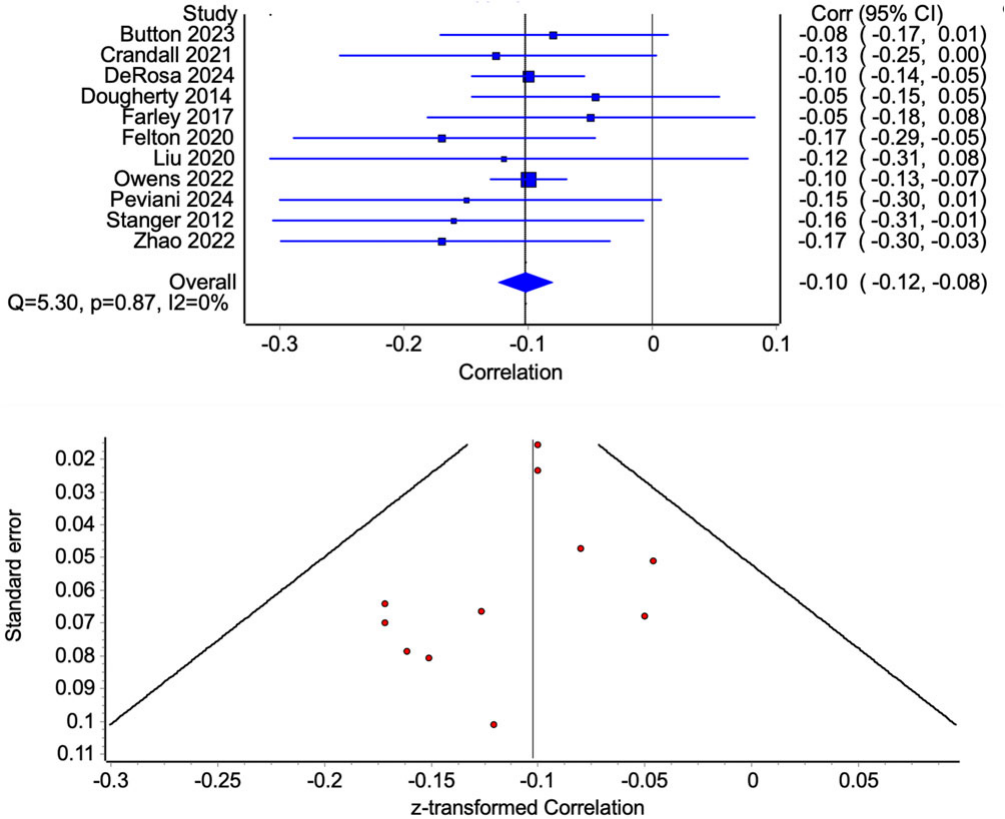
Forest plot of correlations between household income indicators and youth delayed reward discounting (with 95% CI bars) and associated funnel plot examining publication bias.

Eleven studies (each using unique samples) reported negative relations between family/household income or indicators of income and youth delay discounting. The effect sizes of these associations are small, indicated by the correlations ranged from *r* = −0.05 to −0.17 (see Figure 2). Meta-analysis results suggest an overall significant effect of family income on delay discounting (*r* = −0.10, 95% CI = −0.12 to −0.08), also indicating a small effect of lower household income indicators predicting steeper (more problematic) rates of delay discounting in youth. Results also suggest low heterogeneity across studies (*Q* = 5.30, *p* = 0.87, *I* ^2^ = 0%). The pooled result was robust to the exclusion of any individual study, changing by less than 0.01. The funnel plot showed major asymmetry, suggesting possible publication bias.

**Figure 3.**
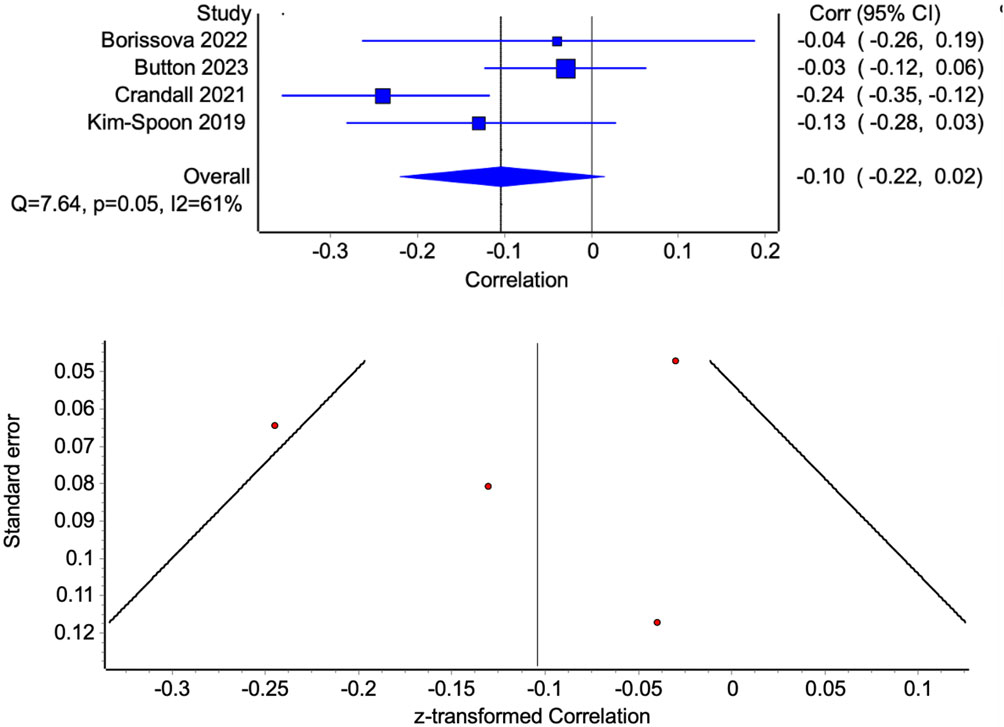
Forest plot of correlations between parental education and youth delayed reward discounting (with 95% CI bars) and associated funnel plot examining publication bias.

Four studies representing unique samples examined parental educational attainment (a widely utilized indicator of socioeconomic status; see Figure 3). A meta-analysis of parental education found pooled correlations comparable to income, but not statistically significant: −0.10 (−0.22, 0.02). Studies were heterogeneous, with an I^2^ of 61% but a non-significant Cochran’s Q (7.64, *p* = 0.05). The pooled result was sensitive to the exclusion of two studies: dropping Button et al. (2023) yielded a pooled estimate of −0.17 (−0.28, −0.06) and dropping Crandall et al. (2021) yielded a pooled estimate of −0.05 (−0.13, 0.02). The funnel plot showed major asymmetry, suggesting possible publication bias.

Other studies included closely related concepts that reflect, or are tied to, family household income. These include three studies that examined the relation between receiving state or federal income assistance and youths’ rate of discounting (Anil et al., 2011; Crandall et al., 2021; Kim-Spoon et al., 2019). None of these studies found significant relations, with bivariate relations ranging from *r* = −0.13 to −0.16. One study examined the correlation between Medicaid status and discounting and found a small, but significant, relation (*r* = −0.16) with parental discounting, but no significant relation with child discounting (*r* = −0.003) (Button et al., 2023). Parent employment had similarly small effects on youth rates of discounting in two studies (Anil et al., 2011; Crandall et al., 2021). These relations were statistically significant in only one of these publications, with Crandall and colleagues (2021) did finding a bivariate relation between children’s discounting and employment prestige (*r* = −0.14). A latent “Family Risk” factor indicated by parent education, family income, and receipt of family income assistance also had a small but significant impact on youth rates of delay discounting one year later (*r* = 0.19) (Kim-Spoon et al., 2019).

Additionally, Farley & Kim-Spoon (2017) examined parents’ perceptions of their financial well-being and youth delay discounting (Farley & Kim-Spoon, 2017). These authors found small and non-significant relations between rates of discounting and parents’ financial satisfaction (*r* = −0.06), financial worries (*r* = −0.06), and perceptions of being “well off” (*r* = −0.07). A latent SES factor created by the authors (including per capita household income and each of the indicators above) was also not significantly related to youth rates of discounting (*r* = −0.07).

### Associations between community-level factors and youth discounting

Only one study evaluated objective indicators of community-level disadvantage. Felton et al. (2024) created a census-derived index including levels of low educational attainment, lone parenthood status, percent of low-income households, and unemployment status derived for participants’ block face. Findings suggest that higher rates of community socioeconomic disadvantage was associated with steeper youth DRD cross-sectionally and over time (from ages 13 – 18). This same study also examined the effect of perceived environmental support on youth DRD. The authors did not find cross-sectional relations with rates of youth DRD but did find that perceived support interacted with the objective measure of socioeconomic disadvantage to predict changes in DRD longitudinally, such that higher levels of perceived support buffered the negative effects of environmental disadvantage over time.

### Associations between parenting and youth discounting

A smaller number of parenting-specific factors have been evaluated in relation to youth rates of delay discounting. Of the three articles (reflecting two unique samples) that evaluated the relation between adverse or harsh parenting and youth discounting, both found significant associations indicating higher rates of harsh discipline were associated with steeper rates of youth discounting. Liu et al. (2020) examined aspects of maladaptive parenting and children’s rates of discounting, finding significant relations with corporal punishment (*r* = −0.25), weekly harsh parenting (*r* = −0.25), and parental neglect (*r* = −0.26). Additionally, Peviani and colleagues (2019) found that harsh parenting was associated with youth delay discounting one year later (*r* = −0.18). A reexamination of the same sample looking at rates of maltreatment and abuse found no significant relations between physical abuse and DRD at any of the five timepoints examined but found that higher rates of neglect predicted steeper DRD at Times 2 – 5 (Peviani et al., 2024b).

### Associations between household instability factors and youth discounting

A small number of studies examined household-specific factors and their association with rates of youth discounting. Two articles reported on household chaos drawn from the same community sample. The first found a relation between chaos (collected at the second wave) and adolescent discounting at the following year (*r* = −0.27) indicating greater levels of household chaos were associated with steeper rates of discounting (Peviani et al., 2019); however, these relations were not significant in previous years (i.e., chaos measured at wave 1 and discounting measured at year 2; Kim-Spoon et al., 2019). Another study found higher rates of household food insecurity were associated with steeper youth delay discounting (*r* = 0.14; Crandall et al., 2021). Finally, a study of household insecurity, specifically being evicted one’s home, was also associated with steeper rates of discounting in a probit model (Anil et al., 2011).

Parental divorce/marital status was also examined in three studies utilizing community samples. Of these, two found significant relations, including parental divorce (Owens et al., 2022, *B* = 1.51, *p* = 6.48E- 11) and not having both parents in the household (Anil et al., 2011, using a probit model) predicting steeper rates of youth discounting. However, parental marital status specifically was not significantly associated with rate of discounting in the latter study (Anil et al., 2011). Similarly, Farley & Kim-Spoon (2017) also did not find that parental marital status was associated with discounting.

### Associations between parent mental and behavioral health and youth discounting

**Figure 4.**
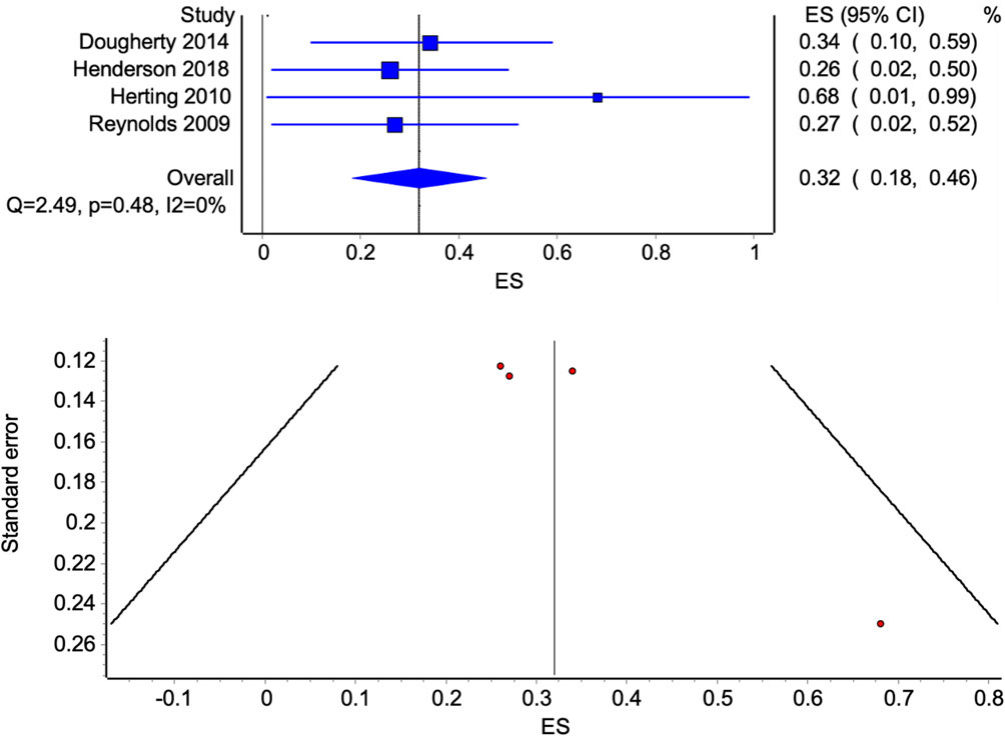
Forest plot of effect sizes of differences between youth with and without family histories of substance use disorders in delayed reward discounting (with 95% CI bars) and associated funnel plot examining publication bias.

Parental substance use and other antisocial behaviors were examined in nine studies, reflecting data from eight independent samples. Of these, four studies (each reflecting independent samples) reported comparable indices of delay discounting among youth with and without family histories of clinically relevant substance use and could be included in a meta-analysis (see Figure 4; Dougherty et al., 2014; Henderson et al., 2018; Herting et al., 2010; Reynolds et al., 2009). Effect size estimates reflecting differences in delay discounting between these groups ranged from *d* = .26 to .68, with an overall medium effect (*d* = .32, 95% CI = .18 to .46) and low heterogeneity among studies (*Q* = 2.49, *p* = 0.48, *I* ^2^ = 0%). Results support the hypothesis that children of parents with substance use disorders demonstrate steeper rates of discounting. Three additional studies also examined differences in DRD among youth with and without family histories of SUD but did not examine bivariate relations and so were not able to be included in the meta-analysis. Dougherty et al. (2015) evaluated trajectories of youth discounting from data collect biannually over three years (data from baseline results of this study were reported in Dougherty et al. (2014) and included in the meta-analysis). The authors found that while baseline levels of discounting statistically differed between groups, trajectories of change in discounting over time did not. In exploratory subsample analyses with youth who did not use substances at any point during the study, baseline differences between groups on rates of discounting were no longer significant, although the authors reported this may be due, in part, to decreases in statistical power to detect effects. Additionally, one study examined differences between children of parents with and without substance use disorder from an inner-city environment (Rodriguez-Moreno et al., 2021). The authors examined a number of differences in characteristics of youth discounting, including when the smaller reward was presented as available immediately versus in two weeks, and when either the smaller or larger reward was presented as the default option. Results suggest youth with a family history of substance use disorder were less likely to choose larger delayed rewards (relative to youth without a family history of substance misuse) when the smaller reward was presented as immediately available. Additionally, participants with a positive family history of substance use disorder increased the rate at which they chose the larger later reward more steeply than youth in the comparison condition as the magnitude of the difference in larger and smaller reward amounts increased. Finally, Paraskevopoulou et al., 2020 examined the effects of having a family history of substance use disorder on rates of DRD among youth with and without ADHD and found neither an effect for ADHD status, family history, nor their interaction in predicting youth DRD.

Two additional studies looked at parent-reported substance use in non-clinical samples. Felton and colleagues (2021) did not find relations with child discounting and maternal self-reported frequency of marijuana use (*r* < .01) in a community sample. An index of family antisociality (including family members substance use and criminal behavior) was also not significantly associated with steeper youth discounting (*r* = 0.15) in another community-based sample (Kim-Spoon et al., 2019).

Three studies examined the role of parental psychopathology and suicidal behaviors on youth discounting. One study of adolescents recruited from the community (Felton et al., 2021) evaluated rates of maternal depressive symptoms, measured continuously, and did not find a significant relation with youth rates of discounting (*r* = 0.15). Conversely, two other studies relations between youth DRD and more clinically significant parent pathology. One study found that having a family history of suicidal behaviors predicted steeper rates of youth discounting (*r* = −0.22) in a clinical sample of children with and without suicide attempts (Bridge et al., 2015). Another study demonstrated that higher rates of parents’ self-injurious behavior was also associated with steeper child DRD (*r* = .28).

### Associations between stressful events and youth discounting

Finally, one study evaluated both dependent (events that a person contributes to) and independent (fateful) life events and youth discounting (Felton et al., 2022). This study found that youths rate of discounting was not significantly related to either dependent (*r* = 0.06) or independent (*r* = 0.04) events; however, and of note, youth discounting did predict greater increases in dependent life events across adolescence.

## Discussion

Disadvantaged environments, defined as the experience of both harshness (including lack of resources) and unpredictability (e.g., exposure to traumatic experiences), have been hypothesized to impact decision-making by reinforcing a propensity to select smaller rewards that are immediately available versus larger rewards that are delayed (e.g., Griskevicius et al., 2011; Martinez et al., 2022). The current paper is the first, to our knowledge, to systematically review these relations among youth aged 18 and under. Findings from this review and meta-analyses suggest some inconsistencies across the literature in terms of the environmental domains that are associated with youth delay discounting but also indicate larger trends associated with children’s rate of delay discounting, detailed below.

### Associations between household indicators of financial resources and youth discounting

The majority of articles in this review included at least one indicator of household resources or socioeconomic status and generally found small effects relations with youth discounting. For instance, results from our meta-analysis of household income were largely consistent and revealed an overall effect of *d* = −0.10, indicating a weak but statistically significant relation between lower levels of family income and steeper (more problematic) rates of discounting. Looking at parent factors, we found similar small and but nonsignificant relation between parent education level (a socioeconomic indicator often linked to household income) and youth discounting. Taken together, these findings support a small impact of household financial hardship on youth decision-making.

### Associations between indicators of household unpredictability and youth discounting

Results suggest potentially stronger relations and more consistent among studies examining specific indicators of household unpredictability and youth decision-making. Specifically, indicators of harsh parenting, including corporal punishment and verbal aggression, were significantly and moderately associated with steeper rates of youth discounting (Liu et al., 2020; Peviani et al., 2019). Food and housing insecurity was also consistently related to more impulsive youth decision-making (Anil et al., 2011; Crandall et al., 2021). Finally, household chaos predicted steeper rates of subsequent youth discounting (Peviani et al., 2019), although these results did not hold in previous waves of data collected from the same sample (Kim-Spoon et al., 2019).

Findings of the effects of other indicators of unpredictability on child discounting appeared to vary based on sample characteristics. For instance, clinically significant parental substance use and psychopathology were significant predictors child delay discounting. Indeed, results from our meta-analysis of studies comparing youth with and without family histories of substance use disorder found an overall small-to-medium effect. Yet, these relations did not hold in studies including community samples, where rates of overall parental pathology may be lower (e.g., Felton et al., 2021). Results of the impact of parental divorce on youth decision-making found mixed results (Anil et al., 2011; Crandall et al., 2021; Farley & Kim-Spoon, 2017; Owens et al., 2022), which may reflect that the impact of parental divorce are complex and do not uniformly predict worse child outcomes (e.g., Brand et al., 2019). Lastly, only study examined an objective (census-derived) indicator of community economic distress, finding that higher levels of neighborhood disadvantage was linked to steeper youth DRD.

Considered together, patterns of findings suggest more consistent relations between rates of delay discounting and environmental factors were apparent between youth delay discounting and both proximal and more severe indicators of community, parenting, and household instability. These results may suggest that indicators of instability that have a more direct impact on the child (i.e., having to move because of the loss of a house, not having enough to eat, living in a disadvantaged neighborhood, or being spanked or hit by a parent) have a larger effect relative to availability of resources (such as household income) which may influence children through other, less direct, pathways (increasing parental stress, etc.). Additionally, exposure to more severe parental pathology (namely clinically significant parental substance use) may exert a stronger impact on youth decision-making relative to less clinically significant exposures (such as occasional parental substance misuse). This may reflect the more destabilizing effects of significant parental pathology and its effects on parent–child relationships and youth development broadly (Connell & Goodman, 2002; Goodman et al., 2011; Kane & Garber, 2004). These findings also complement other research from adult populations suggesting the importance of (financial) stability and the negative impact of sudden financial loss on rates of DRD (Bickel et al., 2016; Haushofer et al., 2013; Mellis et al., 2018), further highlighting the role of perceived stability in future-focused decision-making.

### Clinical implications

These results have a number of potential implications for policy and clinical work. Given the number of public health-relevant outcomes associated with delay discounting, identifying environmental factors with the greatest impact on rates of delay discounting in youth has the potential to shape policy approaches. First, while small, it is important to note there was a consistent relation between household income and rates of delay discounting, suggesting that programs that focus on alleviating family poverty may have a role to play in supporting healthy youth development by improving decision-making. Second, these findings underscore that youth experiencing proximal environmental instability and trauma (housing and food instability, harsh parenting, parental substance use disorder) may have the greatest needs. While speculative, these findings support the potential for improving household stability and reducing harsh parenting to support healthy youth decision-making and wellbeing. Third, findings suggest that associations of environmental domains reflecting harshness and instability with youth delay discounting were stronger in clinical compared to community-based samples, highlighting the importance of additional supports and interventions that may diminish associations between environmental adversity and discounting in clinical samples specifically. Indeed, one study found that differences between youth with positive and negative family histories of substance use disorder were attenuated in a subsample where youth that had not initiated their own substance use, potentially suggesting the role of other protective factors in delaying maladaptive health outcomes and supporting positive decision-making. Moreover, the negative implications of discounting rewards may be further heightened in the context of environments that lack alternative reinforcement (i.e., resource-poor environments). Recent behavioral economics models of substance misuse (such as the contextualized reinforcer pathology model; Acuff et al., 2023) highlight that addiction is influenced by the conjoint effects of a propensity to discount the value of delayed rewards and the availability (or lack of) alternative reinforcers. Youth with elevated rates of DRD who are in resource-poor contexts may, thus, be particularly vulnerable to substance use and related poor health outcomes. These findings therefore also highlight one potential pathway to disrupt intergenerational cycles of mental and behavioral health disorders through improving environments (including social and structural determinants of health), supporting at risk-children and reducing youth DRD in these populations.

### Limitations and future research

By reviewing a variety of environmental indicators of financial and family stability, the current review describes a nuanced pattern of relations between different facets of the environment and youth rates of discounting. However, several limitations to the current review point to avenues for future research. First, there were no studies that directly compared the effect of each of these environmental indicators of risk on youth delay discounting. Large-scale studies with sufficiently powered samples, such as the Adolescent Brain Cognitive Development study, have the potential to look at the relative contributions of indicators of disadvantage in order to identify which predictors, or combination of predictors, are the most impactful on choice behaviors. Second, only one study examined relations between environmental disadvantage and youth delay discounting in a specifically impoverished sample (Liu et al., 2020) and only three studies were drawn from non-US samples (Borissova et al., 2022; Paraskevopoulou et al., 2020; Zhao et al., 2022). Future studies should evaluate these relations in other low-income and international samples to evaluate whether the impact of household income and related environmental factors on youth discounting is exacerbated among more disadvantaged families or in different cultural contexts. Third, we examined these relations among youth samples only. While predicting the impact of environmental disadvantage early in life is important for developing preventative interventions, it may be that economic constructs, including family income, become more salient in adulthood. Thus, future studies should review these relations among adult samples. Fourth, results of this review underscore the need to refine and further examine the operationalization of environmental harshness and scarcity. While many studies relied on parents’ report of annual household income, it seems like that other measures may capture more harmful aspects of environmental disadvantage. Indeed, only one study utilized census-level markers of environmental disadvantage (Felton et al., 2024). Additional studies examining other objective indicators of community disadvantage and disorder may clarify the potential for different community-level intervention (such as neighborhood rejuvenation projects) to improve decision-making among youth. Moreover, this (Felton et al., 2024) was also the only study that examined more positively valanced environmental indicators (i.e., perceived neighborhood support) that could act as potential buffer to the impact of environmental disadvantage on DRD. Fifth, it is important to note that the studies reviewed do not account for genetic confounding or genetic nurture (Bates et al., 2018). Genetic confounding refers to, in this case, the appearance that parents’ DRD is causal of childhood DRD when both are due to common underlying genetic effects. Genetic nurture refers to a scenario where the childhood outcome is indirectly influenced through the rearing environment, which has been shaped by the parents’ genes. Family-based designs, which can directly model these effects, will be crucial for disentangling these relationships in future research. Finally, despite consistent findings that individuals with obesity and substance use disorder demonstrate higher rates of DRD, evidence is mixed suggesting that DRD is a large and unique predictor of these disorders (e.g., Bailey et al., 2021). Thus, future research should evaluate longitudinal models examining mechanistic chains linking environmental influences, DRD, and maladaptive health outcomes.

## Conclusions

The current review examines the relation between youth delay discounting and environmental disadvantage. Findings suggest a small but consistent impact of household income on rates of discounting and larger effects of environmental indicators that youth may experience as more proximal and salient (such as parenting and parental substance use disorders). These results highlight both the need for more research into these associations as well as the potential for targeted policy and intervention efforts to improve youth decision-making and subsequent health outcomes.

## Data Availability

De-identified data, analysis code, and study materials are available upon reasonable request from the corresponding author.

## Pre-registration statement

This systematic review was pre-registered with the International Prospective Register of Systematic Reviews (PROSPERO; Registration ID: CRD42023467088). The registration includes the review objectives, inclusion/exclusion criteria, and planned analytic approach. Any deviations from the registered protocol are described in the manuscript.
